# Mobilising social workers to promote physical activity: a call to action

**DOI:** 10.1186/s12966-026-01981-2

**Published:** 2026-10-05

**Authors:** Brett Smith, Javier Monforte, Tanya Moore, Amarjit Randhawa, Jennifer Airlie

**Affiliations:** 1 Department of Sport and Exercise Sciences, Durham University, Durham, UK; 2 Department d’Educació Física i Esportiva, Universitat de València, València, Spain; 3 Adult Social Care, Essex, UK; 4 Disability Rights UK, London, UK

Social workers (SWs) are a group of social care professionals that have been largely overlooked for the promotion of physical activity (PA). Yet, there are good reasons why they are well-placed and well-versed to promote PA.

First, PA improves physical, mental, emotional and social wellbeing. A key mandate and principle of the social work profession is to advocate *wellbeing* [1] and one cost-effective way to do that is by promoting PA.

Second, advocating for *social justice* and *human rights* is core to social work [1]. Promoting PA is a way to support these mandates and principles whilst contributing to efforts to reduce physical inactivity and improve people’s health. Physical inactivity is a key contributor to poor health. Supporting PA for people whose opportunities for movement and gaining from its health benefits are otherwise constrained by inaccessible environments, poverty, discrimination and wider structural inequalities is an approach grounded in social justice principles. This is why physical activity represents a human right. For instance, PA is a social justice issue because inequities, discrimination, and oppression impact on the quality and levels of participation. Groups like disabled people, Black people, people living in poverty, and people with multiple intersecting identities are disproportionately likely to be inactive and negatively affected by physical inactivity due to health inequities, discrimination, and oppression [2, 3]. SWs can use their social justice mandate and principle to challenge the inequities, discrimination, and oppression that hinder the quantity and quality of PA participation. They can also advocate for people’s rights to benefit from PA. When derived from well-established human rights, such as the right to health, the right to rest and leisure, the right to education, and the principle of non-discrimination, PA becomes an issue of equity and participation that SWs are well placed to champion. For example, SWs can advocate for the provision of local PA-related services and for changes to the built environment that hinder PA.

Third, *ecosocial work* is increasingly mandated and is another principle to guide the social work profession [4]. SWs recognise the interdependence of people, communities and the natural environment. They are called to work proactively with them to address the growing impacts of environmental injustices like climate change that affect human wellbeing, human rights, health equalities, and the earth. A way to uphold eco-social work and the other mandates that interweave with it, whilst simultaneously contributing to the vital new PA *planetary health* agenda [5], is by promoting physical activities that enhance the planet’s health. For example, SWs can raise awareness that physical activity goes beyond just exercise or sport, is about moving wherever, however, and whenever, and movement, including small amounts, can bring multiple benefits. They can promote active sustainable travel and build it into care support planning. SWs can also do ecosocial work by working with communities to create equitable PA green space opportunities, like urban gardening, that are beneficial for human wellbeing and planetary health [5].

Fourth, the social work profession is informed by *different knowledges* that are highly useful for PA promotion and can be showcased through that vehicle [1]. To improve the impact of PA work *interdisciplinary* knowledge and knowledge generated through *co-produced* or *co-designed* research is increasingly called for [3]. Social work is interdisciplinary. It values and integrates different disciplinary knowledge, like from psychology, sociology, ecology, education, and public health. SWs have a long-standing commitment to valuing and integrating the knowledge of people with different lived experiences by working with them in co-produced or co-designed ways. This is highly beneficial for PA promotion because it ensures conversations and interventions are based on what matters to and for people rather than assumptions about what is best for them.

Fifth, SWs practice using *strengths-based*, *person-centred*, *trauma-informed*, and *relational approaches* [1]. Such approaches are central to changing behaviours, making SWs well-placed to support people to make the changes needed to build more PA into their lives.

Sixth, the social work profession has considerable *reach* in terms of who they have regular contact with, for how long, and where [1]. SWs work with people of all ages. They connect with people who could benefit the most from PA promotion. These include disabled people, people with long-term mental ill-health, young carers, people with intersecting identities, children in need, people with alcohol and drug use issues, families in poverty, people seeking asylum, and people living in care homes. The social work profession is particularly well-placed to enable quality PA conversations because, although time pressured, in comparison to many health professionals SWs spend more time with people, discussing a wide range of issues and gaining a deep understanding of how the person lives their life and what matters most to them. A SW visit might last from 15 min to a few hours. Visits can be one-off but, in many cases, will continue over a number of weeks or even longer. The places where SWs connect with people are also varied and align with where PA conversations need to happen as part of routine practice and initiatives like Making Every Contact Count [6]. These include hospitals, people’s homes, hospices, schools, prisons, homeless charities, domestic violence charities, councils, care homes, adoption agencies, children’s homes, and mental health clinics and secure units.

Seventh, another reason why SWs are well-positioned to do PA promotion is that they can be *trusted* by the public for promoting PA [7, 8]. For example, their skills to bear witness to people’s experiences rather than enact epistemic injustice help make SWs trusted professionals for promoting PA [8].

Eighth, social work is a reflective and highly diverse profession that increasingly recognises the importance of supporting its *own workforce* to be more physically active and less sedentary at work [1]. People who are active themselves are more likely to advocate for PA and be taken seriously by others [9].

Ninth, SWs increasingly recognise promotion of PA as part of their *preventative agenda* [1]. PA promotion offers an important vehicle through which social work can further its commitment to prevention. That includes the unnecessary escalation of dependency [1].

Historically, SWs have lacked adequate opportunities to acquire the knowledge, confidence and skills needed to effectively do PA promotion. Policy and leadership support to embed this routinely across the profession has also been lacking. However, there are now signs this is beginning to change. For example, research is emerging that has highlighted the value of SWs in sport settings [10]. Internationally there is the *Alliance of Social Workers in Sports*. Regarding PA more broadly, a co-produced, theory-guided, and evidence-based research programme to train and educate SWs in PA promotion as part of their routine practice was recently produced. This is called *Moving Social Care* (MSC). Beyond training and education, MSC is now impacting on the English social care profession. For example, the English Eastern Region Principal Social Worker Network is now committed to ‘Right to Move’ in which PA promotion is one of three priorities to focus on from 2027. The UK Government’s Chief Social Worker for Adults now wants to embed PA in frontline practice. MSC is also bringing together different professionals, like physiotherapists and exercise professionals, to support interprofessional PA promotion.

By forming a foundation for action, we hope this commentary helps support efforts to mobilise an under-recognised profession that is well-placed to promote PA. SWs can drive change. They should become a core part of global efforts to promote PA.

## Data Availability

No datasets were generated or analysed during the current study.
